# Titanium and Platinum–Fluoroplastic Stapes Prostheses Visualization on Cone Beam Computed Tomography and High-Resolution Computed Tomography

**DOI:** 10.3390/life11020156

**Published:** 2021-02-17

**Authors:** Valerie Dahm, Ursula Schwarz–Nemec, Alice B. Auinger, Michael A. Arnoldner, Alexandra Kaider, Dominik Riss, Christian Czerny, Christoph Arnoldner

**Affiliations:** 1 Department of Otorhinolaryngology, Head and Neck Surgery, Medical University of Vienna, 1090 Vienna, Austria; valerie.dahm@meduniwien.ac.at (V.D.); alice.auinger@meduniwien.ac.at (A.B.A.); dominik.riss@meduniwien.ac.at (D.R.); christoph.arnoldner@meduniwien.ac.at (C.A.); 2 Department of Radiology, Division of Neuroradiology and Musculoskeletal Radiology, Medical University of Vienna, 1090 Vienna, Austria; michael.arnoldner@meduniwien.ac.at (M.A.A.); christian.czerny@meduniwien.ac.at (C.C.); 3 Center for Medical Statistics, Informatics, and Intelligent Systems, Medical University of Vienna, 1090 Vienna, Austria; alexandra.kaider@meduniwien.ac.at

**Keywords:** stapedotomy, piston, computed tomography, cone beam computed tomography

## Abstract

*Objective:* The aim of this study was to evaluate whether stapes prostheses can be visualized with less metal artifacts and therefore more accurately on cone beam computed tomography in comparison to computed tomography imaging. Recent studies have shown that cone beam computed tomography has advantages when imaging metal artifacts. Patients with hearing loss or vertigo, who have undergone stapedotomy, often present a challenge for otologic surgeons. Imaging studies can deliver crucial additional information. *Methods:* A retrospective analysis of imaging studies and clinical data in a tertiary care center were carried out. Forty-one patients with forty-five implanted ears were evaluated in the study. All included patients had been implanted with a platinum–fluoroplastic (n = 19) or titanium (n = 26) piston and subsequently had undergone imaging months or years after surgery for various reasons. Patients underwent computed tomography or cone beam computed tomography of the temporal bone depending on availability. Piston visualization, prosthesis length, vestibular intrusion and audiologic results were compared between the groups. Piston position on imaging studies were compared to intraoperative findings. *Results:* Functional length measurements of all prostheses were carried out with a mean error of −0.17 mm (±0.20). Platinum–fluoroplastic protheses were significantly underestimated in length compared to titanium prostheses. To analyze the material-dependent difference in the measurement errors of the imaging techniques the interaction was tested in an ANOVA model and showed no statistically significant result (*p* = 0.24). The blinded neuroradiologist viewed two implants, both platinum–fluoroplastic pistons, as located outside of the vestibule due to an underestimation of the prothesis length and the missing radiodensity of the lower end of the prosthesis. **Conclusion:** Surgeons and radiologists should be aware of the different types and radiologic features of stapes prostheses and the missing radiodensity of some protheses parts. Cone beam computed tomography is an imaging alternative with a potential advantage of reduced radiation in patients after stapes surgery suffering from vertigo or hearing loss to evaluate piston position.

## 1. Introduction

Patients suffering from otosclerosis can be surgically treated by stapedotomy. When patients suffer from hearing loss or vertigo after stapedotomy in the immediate or delayed setting, surgeons are faced with the question of conservative treatment (i.e., steroids) and revision surgery. Studies have shown that vertigo can be correlated with the length of the prosthesis intruding the vestibule [1,2,3]. Computed tomography (CT) is usually carried out to evaluate prosthesis position and depth of vestibular penetration. Piston lengths especially titanium prostheses are often overestimated on CT imaging [4]. Warren et al. and Hahn et al. questioned the accuracy and therefore the significance of measurement of the depth of vestibular penetration [5,6]. Cone beam computed tomography (CBCT) has shown advantages by reducing metal artifacts [7,8] while exposing the patient to a lower dose of radiation [9]. Nevertheless, until today the imaging method of choice when faced with the question of evaluating stapes prosthesis position seems to be CT [10]. It has previously been described that not all stapes prostheses can be equally visualized on CT [6]; until now, there has been no information in the literature as far as the accuracy and usefulness of different radiologic modalities are concerned. When reviewing the literature and in daily clinical routine, the missing radiodensity of some stapes prostheses and the resulting difficulty of evaluating vestibular penetration is often neglected. The aim of this study was to evaluate the difference of CT and CBCT imaging in titanium and platinum–fluoroplastic stapes prosthesis and evaluating advantages and disadvantages of the two imaging modalities.

## 2. Materials and Methods

### 2.1. Population

A retrospective analysis was carried out to identify patients who underwent stapes surgery and had a high-resolution CT or CBCT of the temporal bone between January 2010 and December 2018. Forty-one patients were included in the study, with 45 ears implanted with stapes prostheses. Imaging studies were performed mainly due to dizziness, hearing loss and pathologies of the contralateral ear. Only patients who were implanted with either a titanium or platinum–fluoroplastic prosthesis, as described below, were included in the study. These two protheses were chosen for analysis since they are used most at our center. Surgeries were performed by several different surgeons. The prosthesis type used depends on the surgeon´s preference. Until 2016, all patients, who were sent for imaging studies, received a computed tomography. After 2016, depending on availability, either a CBCT or a CT were performed. Informed consent was obtained from all subjects involved in the study.

### 2.2. Data Collection and Measurements

Patient details including audiometric data, type of prosthesis and length were collected from medical records. All CT and CBCT examinations were anonymized and randomly presented to two different neuroradiologists, who were not aware of any clinical data. For all prostheses and both imaging groups total prosthesis length, functional length, vestibular penetration and overall prosthesis position were evaluated. All measurements were performed with a picture archiving and communication system (AGFA Health care, IMPAX) integrated measuring tool. Before measuring, MPR (multiplanar reformation) images in axial, sagittal and coronal views were reconstructed with an integrated software tool to depict the implant in its full length. Additionally, to the interrater reliability, one of the radiologists conducted all measurements at two different time points to assess for intrarater reliability.

**Figure 1:**
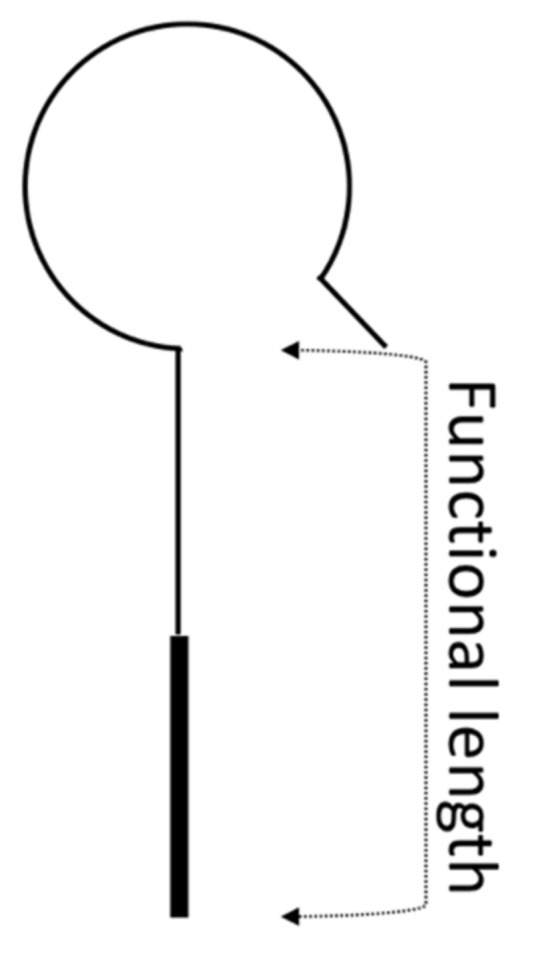
Illustration of a prosthesis and indication of the part of the prosthesis measured when using the term “functional length” according to the manufacturers.

For titanium pistons (fully radiopaque), total prosthesis length was measured from the proximal end to the distal end of the prosthesis. Furthermore, functional length was measured from the base of the titanium loop to the distal end of the prosthesis (Figure 1). Vestibular penetration depth was measured from the underside of the footplate to the distal end of the prosthesis.

**Figure 2:**
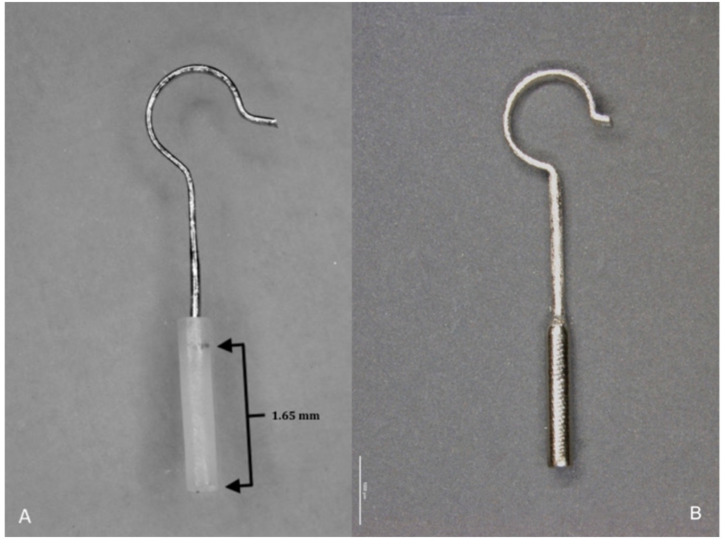
(**A**) Platinum–fluoroplastic piston. Note that the platinum wire reaches into the fluoroplastic portion of the piston. Piston length 4.5 mm. The non-radiopaque (platinum free) fluoroplastic portion measures 1.65 mm (irrespective of prothesis length). (**B**) Titanium piston. Piston length 4.5 mm.

**Figure 3:**
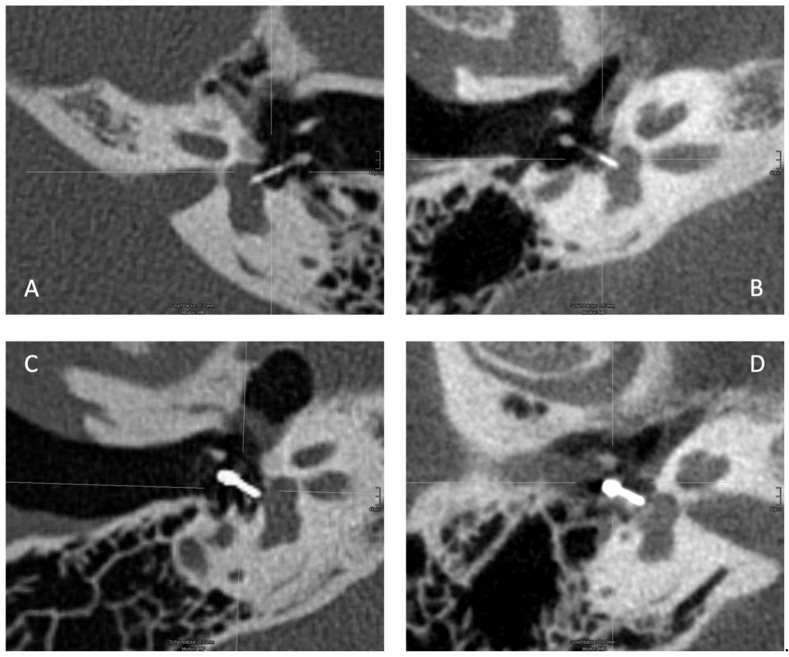
(**A**) Titanium piston on CT. (**B**) Titanium piston on CBCT. (**C**) Platinum–fluoroplastic piston on CT. (**D**) Platinum–fluoroplastic piston on CBCT. (CT—computed tomography, CBCT—cone beam computed tomography).

In contrast, the platinum–fluoroplastic prosthesis is not fully radiopaque: The loop and part of the stem of the prosthesis used in this study is made of platinum, the rest of the stem (the thicker part) penetrating the footplate (Figure 2) is made of fluoroplastic, which is not visible on CT or CBCT. The fluoroplastic portion (without platinum core, Figure 2) measures 1.65 mm irrespective of prothesis length according to the manufacturer and was added to all measurements. Vestibular penetration depth was measured from the underside of the footplate to the calculated end of the prosthesis, adding 1.65 mm to the actual end of the prosthesis. Prosthesis position was evaluated on axial images by assessing the location of the distal end within the vestibule as well as the location of the entire prosthesis within the tympanon. Examples of both protheses materials on CT and CBCT are shown in Figure 3.

Prosthesis material and hearing outcomes were correlated using pure tone audiogram. Hearing tests were performed preoperatively one day up to a maximum of three months preoperatively. The postoperative hearing was tested in general four to six months after surgery. The mean improvement of air-bone gap (ABG) was calculated in decibels as the difference between the preoperative and the postoperative air conduction (AC) and bone conduction (BC) thresholds at four frequencies (0.5, 1, 2 and 4 kHz). If patients underwent revision surgery the preoperative imaging results were correlated to intraoperative findings.

### 2.3. Prostheses Types

All patients included in the study were either implanted with a titanium stapes piston (Kurz) or a platinum–fluoroplastic prosthesis (Gyrus, ACMI). Prostheses used ranged from 4.25 to 6 mm functional length. Functional lengths of these two types of prostheses are measured as illustrated in Figure 2.

### 2.4. Computed Tomography and Cone Beam Computed Tomography

Computed tomography examinations were performed using a standard protocol to assess the temporal bone using a 64-detector row scanner (Philips Brilliance 64, Philips Medical Systems, Best, the Netherlands). Parameters used were as follows: 140 kV, 200 mAs, pitch 0.3, 20 × 0.6 collimation, 768 × 768 matrix, 0.75 s rotation time and slice thickness of 0.67 mm.

Cone beam computed tomography examinations were performed on a Kavo 3D Exam scanner (Kavo Dental, Charlotte NC, USA). Parameters used were 120 kV, 7 s exposure time, 1280 × 1280 matrix and slice thickness 0.13 mm.

### 2.5. Statistical Analysis

Continuous variables are described by mean (± standard deviation) in case of normally distributed data, the median (quartiles) are given for non-normally distributed continuous variables. Normality of distributions was checked using graphical methods. No statistical tests were used to check for normality since these tests (e.g. the Kolmogorov Smirnov test) are flawed, having low power in small samples and—on the other hand—presenting significance for irrelevant deviations in large samples. Differences in the evaluation of the two different radiologists were calculated to describe the interrater reliability, the differences between the two ratings of the same radiologist to assess the intrarater reliability. The difference between the measured length of the prosthesis and the actual length was considered as the primary outcome of interest (measurement error = (measured length) - (actual length)). To calculate this primary outcome measure we randomly selected one evaluation of one of the two raters for each observation. A Bland-Altman plot [11] is used to visualize the relation between the measurement error and the actual prosthesis length. The two-sample t-test was used to compare normally distributed variables between groups of patients and the non-parametric Wilcoxon rank sum test was used in case of non-normally distributed data. A two-way analysis of variance (ANOVA) model, including the factors material, imaging technique and the interaction term, was used to test for a material-dependent difference in the measurement error due to the imaging techniques. Two-sided *p*-values < 0.05 were considered as indicating statistical significance. The software SAS (SAS Institute Inc. (2016), Cary, NC USA) was used for statistical analyses.

## 3. Results

Forty-one patients with 45 stapes prostheses were included in the study. Twenty-one left ears and 24 right ears. There were 16 male ears and 25 females. Twenty-four prostheses were evaluated on CT imaging, while 21 were measured on CBCT. Twenty-six titanium prostheses were evaluated and 19 platinum–fluoroplastic pistons. Patient details are presented in Table 1 and Table 2.

### 3.1. Prostheses Measurements

**Figure 4:**
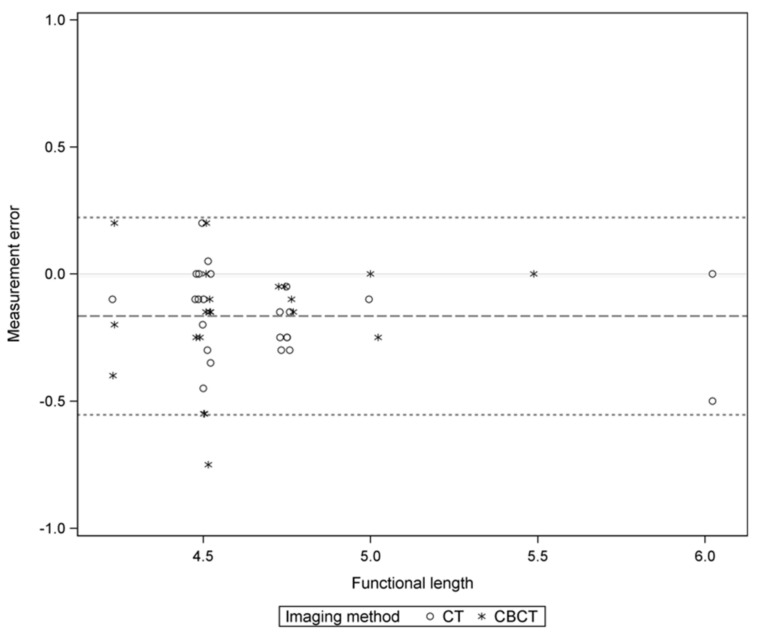
Measurement errors of the functional length are shown as circles and stars. Circles represent measurement on CBCT, stars measurements on CT. Values above 0.0 are overestimated measurements, below 0.0 underestimated measurements. The dashed line represents the mean measurement error. The dotted line represents the margin of the standard deviation.

**Figure 5:**
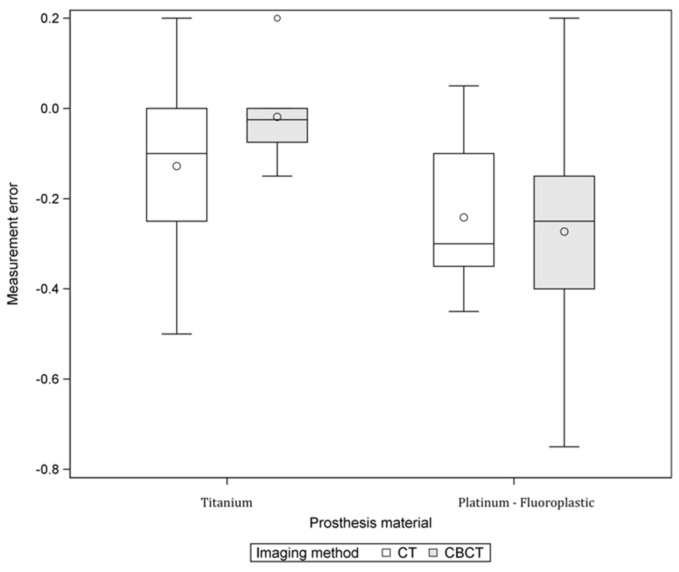
Measurement errors of the functional length are presented in four groups according to prosthesis material and imaging modality. Horizontal lines denote the median, circles indicate the group mean.

On both, CT and CBCT, functional length measurements of all prostheses were carried out with a mean error of −0.17 mm (±0.20). Both prostheses were measured with a mean error of −0.16 (±0.17) on the CT and −0.18 (±0.23) on the CBCT (t-test, *p*-value = 0.74), shown in Figure 4. Titanium prostheses were measured with a mean error of −0.09 (±0.15) and platinum–fluoroplastic prostheses with a mean error of −0.26 (±0.22). The platinum–fluoroplastic prostheses were significantly stronger and more often underestimated (t-test, *p*-value = 0.004). Platinum–fluoroplastic protheses were significantly underestimated in length compared to titanium prostheses. In total, prosthesis length (functional length) was overestimated in 4 (9%) cases and underestimated in 34 cases (76%). Details are depicted in Table 1 and shown in Figure 5. Mean measurement error according to each imaging method and prosthesis material are shown in Table 3.

To analyze the material-dependent difference in the measurement errors of the imaging techniques the interaction was tested in an ANOVA model and showed no statistically significant result (*p* = 0.24). The median (quartiles) vestibular intrusion was 1.3 (1.0–1.6) in titanium implants and 1.7 (1.4–1.7) in platinum–fluoroplastic prostheses (Wilcoxon rank sum test, *p* = 0.027) with no significant difference between CT and CBCT measurements (Wilcoxon rank sum test, *p* = 0.630).

Interrater reliability was high with a mean measurement difference of 0.049 (±0.17) when measuring the functional length. Repetitive assessments of the same reader showed a mean difference of 0.004 (±0.15) when measuring the functional length.

### 3.2. CT and CBCT Accuracy

Six patients underwent revision surgery at the study center. Five of these patients had a CT preoperatively and one a CBCT. The CBCT showed a dislocated prosthesis which was confirmed intraoperatively. In three CT reports the prosthesis was considered to reach too far into the vestibule. All of these patients suffered from conductive hearing loss, none of them complained of vertigo. Hearing improved after revision surgery (including placing a new prosthesis) in two of these patients. One CT showed a pneumolabyrinth five years after surgery. The last CT showed a good position of the piston. Intraoperatively, a loose contact to the incus could be found to be the cause of hearing loss.

The blinded neuroradiologist evaluated two prostheses of patients who did not undergo revision surgery as dislocated, in the CBCT. Two of the prostheses, both platinum –fluoroplastic pistons, were thought not to penetrate the vestibule. Since patients had good hearing results in terms of an adequate ABG at time of imaging, actual dislocation (prosthesis not reaching into the vestibule) of the stapes prosthesis can be ruled out.

### 3.3. Hearing Results

The median (quartiles) preoperative ABG, were 40 (35–55), 35 (25–45), 30 (20–35), 15 (0–20) and 30 (15–35) dB HL at 0.25, 0.5, 1, 2 and 4 kHz, respectively. The median (quartiles) postoperative ABG were 15 (10–20), 10 (0–15), 10 (5–15), 0 (0–5), 10 (5–20) dB HL at 0.25, 0.5, 1, 2 and 4 kHz, respectively.

The mean (± standard deviation, range) improvement of ABG changes of all patients were 30 dB HL (±14, 0–50), 27 dB HL (±16, 0–55), 20 dB HL (±11, 0–40), 11 dB HL (±11, 15–35), 16 dB HL (±14, 15–40) at 0.25, 0.5, 1, 2 and 4 kHz.

There was no significant difference between the groups in the ABG changes when comparing titanium and platinum–fluoroplastic prostheses. The mean (± standard deviation) improvements at 0.25, 0.5, 1, 2 and 4 kHz were 29 (±14), 28 (±18), 21 (±9), 12 (±9) and 17 (±14) dB in the titanium group and 30 (±15), 26 (±14), 20 (±10), 9 (±11) and 15 (±13) dB in the platinum–fluoroplastic group with a *p*-value of 0.8, 0.8, 0.7, 0.4, 0.7 respectively.

## 4. Discussion

In this study, a comparison of stapes prostheses visualization on CT and CBCT imaging was carried out. No statistically significant difference of imaging modality could be found for both prosthesis materials. The results in this study show that both CT and CBCT can be used to assess position of stapes prostheses. The length of the prostheses was measured with a mean error of −0.17 mm. Titanium prostheses were measured with a mean error of −0.09 mm and platinum–fluoroplastic prostheses with a mean error of −0.26 mm. Especially for the titanium prosthesis a high accuracy could be achieved. The platinum–fluoroplastic prostheses were significantly stronger and more often underestimated (*p*-value = 0.004).

The blinded neuroradiologist viewed two implants, both platinum–fluoroplastic pistons, as located outside of the vestibule due to an underestimation of the prothesis length and the missing radiodensity of the lower end of the prosthesis. It is important to be aware of the length of the prosthesis and the extent of radiolucency to avoid false diagnosis of prosthesis dislocation. Our results show that the material must be taken into consideration and must be known to the radiologist judging the piston position when undertaking imaging studies. In platinum–fluoroplastic prostheses a partial missing radiodensity can be expected as also briefly mentioned in a study by Zaoui et al. [12]. Information on the length of the prosthesis and the extent of radiolucency should be provided by manufacturers and marked in implant passes. A further hazard when assessing implant position and vestibular intrusion in imaging studies is that functional length is not always measured as depicted in Figure 1. Some companies measure functional length as total prosthesis length, and some measure up to the center of the loop. The prosthesis used in this study measure functional length as depicted in Figure 1.

The length of vestibular intrusion and its significance has been analyzed in different studies. If the prosthesis is placed too deep into the vestibule patients can experience vertigo. Intravestibular prosthesis depth have been reported to be safe in a range from 0.5 to 1.7 mm at different areas of the footplate [13]. Warren et al. performed CT in eight cadaveric temporal bones and could show that the depth of vestibular penetration was overestimated in stainless steel prosthesis and underestimated in fluoroplastic pistons [6]. In the present study, both prostheses were underestimated more often than overestimated when measuring functional length. This fact has been shown in studies on protheses with fluoroplastic [6]. In contrast, titanium prostheses have been shown to be rather overestimated. In a study by Fang et al. these pistons were overestimated by 0.10 mm [14].

Bozzato et al. analyzed six fresh human temporal bones and showed that titanium pistons were overestimated on different CT imaging techniques by a mean of 0.13 mm. Four different observers measured the prostheses with one observer consistently underestimating them [4]. Three-dimensional volume rendering with CT has been shown to result in high accuracy for cochlear implant position imaging, which might be also be an option for stapes prosthesis visualization in the future [15].

Although it is difficult to compare radiation doses of CBCT and CT due to technical differences [7] several studies have shown that CBCT imaging has less radiation exposure for patients than CT [9]. A study conducted on human temporal bones concluded that CBCT is a suitable alternative to high-resolution CT to assess middle ear anatomy and implanted prostheses due to a low sensitivity for metallic artifacts and low radiation dose [7]. A further, more recent study by Guyader et al. showed a 4.3 to 9.6-fold lower radiation dose of CBCT compared to CT in temporal bone images [16]. Additionally, CBCT has the advantages of less investment costs, easy handling and smaller necessary space. A disadvantage of CBCT imaging is that most patients are seated while studies are performed, which leads to a higher rate of movement artifacts. This argument is underlined by the results of our study. There were two CBCT studies with significant movement artifacts. Despite movement artifacts repeat imaging was not necessary in these two patients.

Several studies have tried to find the ideal prosthesis for best possible audiometric results.

Up to now, no significant differences could be found when using different materials and shapes [17,18]. The same results were shown in this study with no difference in audiometric outcome between the two different prostheses groups. Decision on prosthesis shape and material is therefore mainly based on the preference of the surgeon.

## 5. Conclusions

Cone beam computed tomography is an imaging alternative with a potential advantage of reduced radiation in patients after stapes surgery suffering from vertigo or hearing loss to evaluate piston position. It is important for surgeons and radiologists to be aware of the type and length of prosthesis implanted as platinum–fluoroplastic protheses were significantly underestimated in length compared to titanium prostheses.

## Figures and Tables

**Table 1:** Patient details according to different imaging modalities. CT—computed tomography, CBCT—cone beam computed tomography, y – years. Numbers (unless in parenthesis) reflect number of cases.

Imaging Modality	CT	CBCT
Titanium	18	8
Platinum–fluoroplastic	6	13
Mean age at time of surgery	46 y (28–75 y)	46 y (31–70 y)
Images with movement artifacts	0	2
Overestimation of functional length	2 (8.3%)	2 (9.5%)
Correct measurement of functional length	4 (16.7%)	3 (14.3%)
Underestimation of functional length	18 (75.0%)	16 (76.2%)
Total	24	21

**Table 2:** Patient details of the titanium prosthesis and the platinum–fluoroplastic group. T—titanium prosthesis, PF—platinum–fluoroplastic prothesis, y—years, dB—decibel, HL—hearing level. Numbers (unless indicated otherwise) reflect number of cases.

Prosthesis Material	T	PF
Mean age at time of surgery	47 y (28–75)	44 y (31–61)
Median vestibular intrusion	1.3 mm	1.4 mm
Prosthesis does not seem to reach into vestibule in imaging	0	2
4.50 mm functional prosthesis length	12	13
Mean ABG before surgery	30.0 dB HL	29.0 dB HL
Mean ABG after surgery	10.8 dB HL	9.4 dB HL
Overestimation of functional length	2 (7.7%)	2 (10.5%)
Correct measurement of functional length	7 (26.9%)	0 (0.0%)
Underestimation of functional length	17 (65.4%)	17 (89.5%)
Total	26	19

**Table 3:** Mean measurement error of functional length plus standard deviation (given in parentheses) according to each material (T—titanium, PF—platinum–fluoroplastic) and both groups together (total) for both imaging techniques (CT—computed tomography, cone beam computed tomography) and for each individual imaging method.

Mean Functional Length Measurement Error	T	PF	Total
Independent of imaging	−0.09 (±0.15)	−0.26 (±0.22)	−0.17 mm (±0.20)
CT	−0.13 (±0.15)	−0.24 (±0.18)	−0.16 (±0.17)
CBCT	−0.02 (±0.10)	−0.27 (±0.20)	−0.18 (±0.23)

## Data Availability

The data that support the findings of this study are available from the corresponding author upon request.
